# Hospital for Women, Soho Square

**Published:** 1893-05-27

**Authors:** 


### HOSPITAL FOR WOMEN, SOHO SQUARE.

The Duke of Westminster presided over the fiftieth annual meeting of the governors and subscribers of the above hospital, which was held yesterday in the board room of that institution, 30, Soho Square, W. Amongst those present were the Countess of Romney, Lady Catherine Cust, the Hon. Mrs. Robert Marsham, the Hon. Catherine Somerset, Sir Rutherford Alcock, K.C.B., Dr. C. H. Carter, Dr. Smith, Mr. David Cannon (secretary), &c. The report showed that the number of in-patients admitted during the year was 580, being a reduction of ninety from the previous year, which was partly due to an outbreak of influenza amongst the nursing staff, and partly to the whole hospital being closed during the month of August for alterations. Through the agency of the Samaritan funds forty-nine patients were sent to convalescent homes, and thirty-seven received gifts of money on leaving the hospital. The year's receipts from all sources amounted to £21,360, but they mainly consisted of the exceptionally large sum in respect of legacies. The average ordinary income fell short of the necessary expenditure by £2,000 annually. The committee desired to acknowledge very gratefully the grants by the Hospital Sunday and Saturday Funds. The amount received from in-patients payment was £828 7s, which was less than half of what had been received in a twelvemonth in former years, the reduced amount being due to the committee having made available a large proportion of beds in the hospital for free cases. The Chairman, in moving the adoption of the report, remarked that a particularly encouraging feature was the large amount that had been received in legacies. Another cause for congratulation was that the committee had decided to spend a considerable sum upon improvements which were very urgently required in the building itself. It was also proposed to extend the building operations of the hospital, thereby affording larger accommodation for the out-patients, and giving additional accommodation for a larger staff of nurses. By those means they provided not only increased comfort, but afforded the facilities for obtaining more light and air, which were of the utmost importance. He hoped they would do their utmost to obtain additional subscribers, especially as so large a sum had been lost by the death of many friends, and the discontinuance of others of their customary support. At the conclusion of the dinner the Duke of Sutherland and the Bishop of Rochester were elected vice-presidents of the hospital.

